# Stochastic assembly of biomacromolecular complexes: impact and implications on charge interpretation in native mass spectrometry†

**DOI:** 10.1039/d3sc03228k

**Published:** 2023-08-17

**Authors:** Victor Yin, Paul W. A. Devine, Janet C. Saunders, Arjan Barendregt, Fiona Cusdin, Alexandra Ristani, Alistair Hines, Sam Shepherd, Marcin Dembek, Claire L. Dobson, Joost Snijder, Nicholas J. Bond, Albert J. R. Heck

**Affiliations:** a Biomolecular Mass Spectrometry and Proteomics, Bijvoet Center for Biomolecular Research and Utrecht Institute for Pharmaceutical Sciences, Utrecht University Padualaan 8, 3584 CH Utrecht The Netherlands a.j.r.heck@uu.nl; b Netherlands Proteomics Center Padualaan 8, 3584 CH Utrecht The Netherlands; c Analytical Sciences, Biopharmaceutical Development, R & D, AstraZeneca Granta Park Cambridge UK; d In Vivo Expressed Biologics, Discovery Sciences, R & D, AstraZeneca Granta Park Cambridge UK; e Purification Process Sciences, Biopharmaceutical Development, R & D, AstraZeneca Granta Park Cambridge UK

## Abstract

Native mass spectrometry is a potent method for characterizing biomacromolecular assemblies. A critical aspect to extracting accurate mass information is the correct inference of the ion ensemble charge states. While a variety of experimental strategies and algorithms have been developed to facilitate this, virtually all approaches rely on the implicit assumption that any peaks in a native mass spectrum can be directly attributed to an underlying charge state distribution. Here, we demonstrate that this paradigm breaks down for several types of macromolecular protein complexes due to the intrinsic heterogeneity induced by the stochastic nature of their assembly. Utilizing several protein assemblies of adeno-associated virus capsids and ferritin, we demonstrate that these particles can produce a variety of unexpected spectral appearances, some of which appear superficially similar to a resolved charge state distribution. When interpreted using conventional charge inference strategies, these distorted spectra can lead to substantial errors in the calculated mass (up to ∼5%). We provide a novel analytical framework to interpret and extract mass information from these spectra by combining high-resolution native mass spectrometry, single particle Orbitrap-based charge detection mass spectrometry, and sophisticated spectral simulations based on a stochastic assembly model. We uncover that these mass spectra are extremely sensitive to not only mass heterogeneity within the subunits, but also to the magnitude and width of their charge state distributions. As we postulate that many protein complexes assemble stochastically, this framework provides a generalizable solution, further extending the usability of native mass spectrometry in the characterization of biomacromolecular assemblies.

## Abstract 2

An interpretative framework for analyzing by spectral simulations high resolution native mass spectra of protein assemblies is proposed. It reveals that the spectral appearances of heterogeneous assemblies can be misleadingly simple.

## Graphical abstract

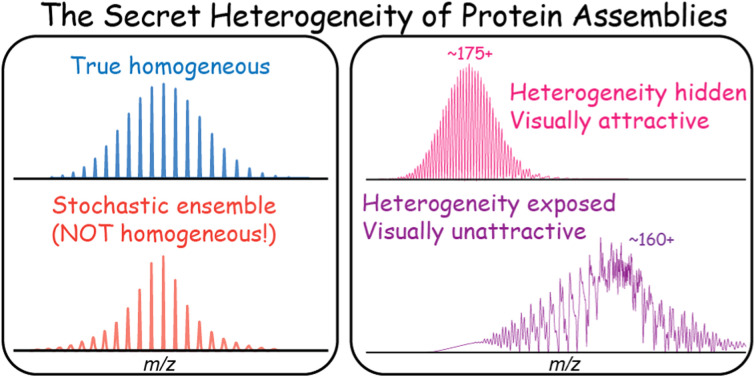

## Introduction

The advent of high-resolution native mass spectrometry (native MS) has enabled the structural characterization of intact proteins and their associated complexes.^1–5^ Information such as subunit stoichiometries, oligomeric states, size distributions, or cargo loading ratios can be readily extracted from a native MS measurement.^6,7^ Nowadays, several mass analyzers are capable of transmitting ions well into the megadalton mass regime, allowing systems as large as intact viral capsids to be successfully measured.^8,9^

Typically, the mass of an analyte is not directly measured in mass spectrometry but determined indirectly from the mass-to-charge (*m*/*z*) positions of peaks in the spectrum. In an electrospray-based native MS experiment, most analytes exhibit a charge state distribution (CSD) in the spectrum, *i.e.*, a collection of peaks with the same mass, but a series of charge values.^10^ The correct assignment of the CSD charge values is critical for successful mass determination. For larger macromolecules which lack sufficient resolving power for isotopic resolution, charge values must be indirectly inferred, *e.g.*, by using the spacing between adjacent *m*/*z* peaks.^11^ Various algorithms and numerical approaches have been developed to facilitate the charge state inferencing task, each with their own strengths and shortcomings, as recently extensively reviewed.^12^

A key assumption underlying virtually all deconvolution approaches is that the peaks observed in a native mass spectrum can be directly mapped to a given mass of a specific charge state. While this is clearly the case for homogeneous, monodisperse smaller proteins, the picture becomes more complicated when mass heterogeneity (*e.g.*, due to post-translation modifications, polydispersity, isoform variations, *etc.*) is introduced. As the mass heterogeneity of the sample increases, the number of expected peaks in the native mass spectrum also increase. Eventually, this heterogeneity will cause peaks to coalesce in the *m*/*z* domain as a single broad, unresolved signal. When this occurs, standard deconvolution (and thus mass assignment) is impossible since the observed signals can no longer be uniquely mapped to a charge state. This binary classification results in the commonly adapted, strictly black-and-white interpretation of heterogeneity, with assemblies being considered either (1) sufficiently homogeneous to produce well-resolved CSDs, or (2) sufficiently heterogeneous to yield essentially featureless, broad distributions where no resolved signals are observed. A corollary of this view is that if a system of unknown complexity does yield resolved ion signals, the system is assumed to be (relatively) homogeneous.

It is interesting to ask whether this assumption correlating system and spectral heterogeneity is generally valid. In other words, can scenarios exist where extremely heterogeneous systems somehow yield “well-resolved” mass spectra? Earlier work from our lab by Snijder *et al.*^6^ and Wörner *et al.*^13^ have suggested that this occurs in the seemingly special case of adeno-associated virus capsids (AAVs), where native mass spectra can paint an erroneous, misleadingly picture regarding the true heterogeneity and masses of the system. AAVs are small, non-pathogenic, single-stranded DNA viruses that are incapable of replication in the absence of a helper virus.^14^ These favourable properties have resulted in increasing interest in the use of recombinant AAVs as a gene therapy vector due their capability to be packed with a therapeutic human gene of interest (GOI).^15–17^ AAVs form 60-mer capsids comprised of three major capsid protein isoforms: VP1, VP2, and VP3 (81, 66, and 59 kDa, respectively).^18^ Primarily based on measurements at the subunit level by denaturing liquid chromatography, capillary electrophoresis, and/or gel electrophoresis, intact AAV capsids were generally assumed to hold ∼5 : 5 : 50 protomers of VP1, VP2, and VP3 per capsid at a more or less fixed subunit stoichiometry. Instead, Wörner *et al.* proposed that AAV capsids assemble stochastically from the available VP monomers, producing in theory an extremely heterogeneous population of 1891 different stoichiometries. Such a complex mixture of stoichiometries and masses would never be expected to generate charge-state resolved mass spectra, seemingly in conflict with the well-resolved native mass spectral appearances of AAVs in several reports.^6,13,19^ We earlier argued that these well-resolved appearances could be due to a mathematical coincidence, as the *m*/*z* distance between adjacent charge states of one AAV capsid happens to be approximately equal to the *m*/*z* distance arising from mass differences between several different VP isoforms, thus leading to a scenario where signals from different species become concentrated in certain *m*/*z* values, yielding a final “false”, albeit well-resolved, appearance of a triplet of CSDs.^6,13^ As such, the fairly simplistic native mass spectra of these AAVs belie their extreme underlying heterogeneity arising from stochastic assembly, and provide a good example of a case where the implicit correlation between spectral and sample heterogeneity can be false. To the best of our knowledge, further examples of this have never been provided outside of these canonical AAVs.

Here, in several steps we critically and systematically evaluate, validate, and extend this interpretive framework of spectral heterogeneity beyond the previously reported AAV spectra, with the aim to provide a correct interpretation of the composition of any macromolecular assembly containing multiple copies of distinct subunits. Firstly, we demonstrate that the canonical AAVs can produce several, highly divergent spectral appearances of varying levels of apparent heterogeneity, seemingly in contrast to the previously-reported well-resolved peaks. By combining high-resolution native MS in parallel with single particle charge detection mass spectrometry and sophisticated spectral simulations, we reveal that these peculiar spectral features arise from the interplay of variations in both the magnitude and width of the experimental CSDs, as well as critical areas along the *m*/*z* axis which allow for stochastic assembly-induced signal concentration effects. Secondly, using a variety of engineered virus-like particles (VLPs) of defined heterogeneity, we demonstrate that native mass spectra of heterogeneous particle populations can appear deceptively like a single homogeneous CSD, but with *m*/*z* positions that are distorted from their “expected” values. Critically, naïve charge state inferencing of these features can lead to substantial errors in the extracted mass (up to 5%), alongside misidentification of the underlying heterogeneity in these systems. Finally, we demonstrate that these spectral phenomena are not unique to viral capsids and may have gone unnoticed so far in other, well-studied protein complexes, as we show here for apoferritin. Our work provides a novel framework contributing to the unambiguous interpretation of high-resolution native mass spectra from any macromolecular assembly.

## Results

### High resolution native MS of AAVs

**Fig. 1:**
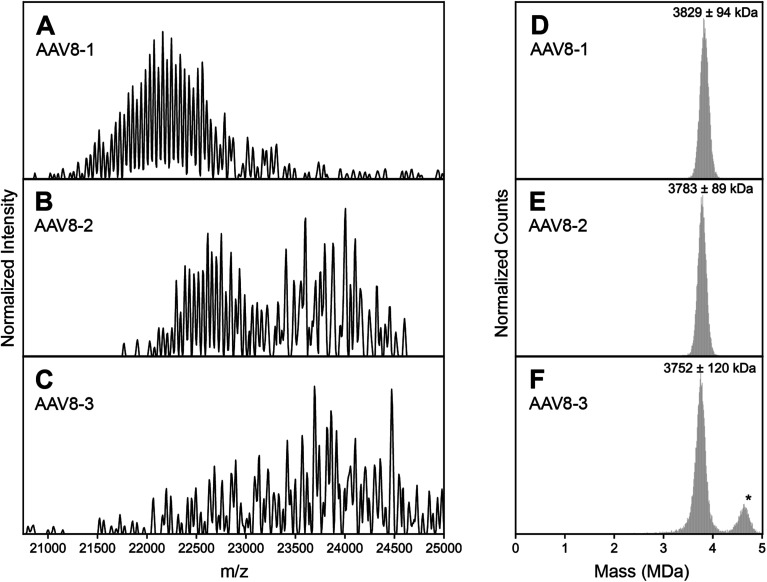
Experimental high resolution native mass spectra of three batches of AAV8 capsids recorded under identical conditions. (A–C) High resolution native mass spectra recorded for three AAV8 preparations, termed AAV8-1, AAV8-2 and AAV8-3. These three preparations result in drastically different spectral appearances, with resolving power not only appearing to be sample dependent, but also *m*/*z* dependent. (D–F) Native Orbitrap-based charge detection mass histograms of the three AAV8 preparations, with the apparent mass of each capsid annotated. In contrast to their divergent native mass spectral appearances, the Orbitrap-based CDMS histograms are very similar. An additional peak (*) is observed in AAV8-3, which corresponds to a population of capsids that contain a transgene. Further information on each capsid sample can be found in Table S1.†

The basis for this study started when we collected high-resolution native mass spectra of three independent batches of recombinant AAV serotype 8 capsids (termed AAV8-1, AAV8-2 and AAV8-3, Fig. 1). Of these three preparations, AAV8-1 exhibited a seemingly well-resolved spectral appearance, similar to those reported earlier for other AAVs (Fig. 1A).^13,19^ However, AAV8-2 and AAV8-3 displayed native mass spectral appearances substantially different (Fig. 1B and C). In the AAV8-2 spectra, a seemingly “high-resolution” segment is observed at *ca.* 23 000 *m*/*z*, alongside a putative second segment at *ca.* 24 000 *m*/*z* that is however much less resolved. For AAV8-3, no high-resolution segment is observed at all, with unresolved signals at *ca.* 24 000 *m*/*z* appearing to comprise the entirety of the mass spectrum.

At a first glance, one could conclude that AAV8-2 and certainly AAV8-3 are “less pure” compared to AAV8-1. A typical, often-invoked rationalization of these spectral appearances would be that AAV8-2 and AAV8-3 are simply (partly) misassembled and/or degraded. To validate the integrity and purity of these three AAV8 preparations, we further analyzed the preparations using several orthogonal methods. We first used single particle Orbitrap-based charge detection mass spectrometry (Orbitrap-based CDMS).^20–22^ Unlike the typical, ensemble native MS experiment, Orbitrap-based CDMS is capable of extracting mass information from even extremely heterogeneous systems by directly extracting charge information from individual ions *in lieu* of any ensemble charge state inferencing.^9,23–25^ The mass histograms obtained *via* Orbitrap-based CDMS for all three AAV8 preparations were all in line with the expected mass of purified AAV capsids (Fig. 1D–F), with no masses corresponding to partially assembled capsids or degradation products. We additionally imaged each AAV8 preparation using negative stain transmission electron microscopy (Fig. S3†), further confirming that the assembled capsids form the majority of species in all three samples. Therefore, we rule out that the observed unusual features in the high-resolution native mass spectra of AAV8-2 and AAV8-3 are due to defects and/or impurities and thus must arise from correctly assembled capsids.

**Fig. 2:**
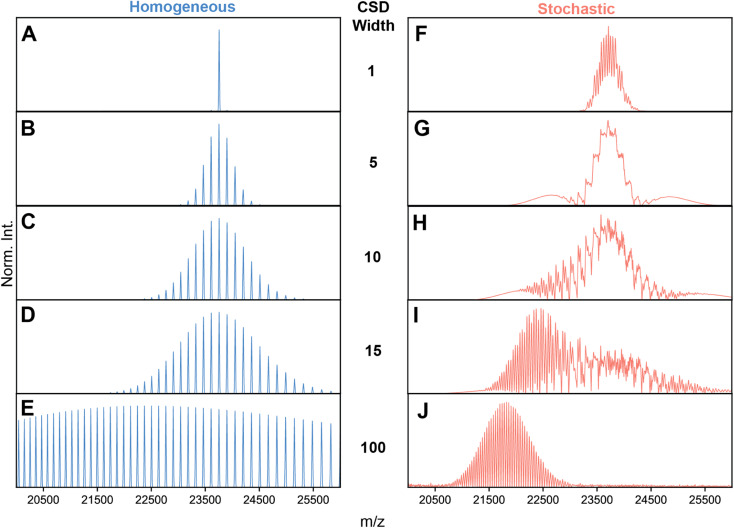
Simulations of AAV high-resolution native mass spectra at pre-set capsid heterogeneities and CSD width. (A–E) Simulations of an idealized, homogeneous (*i.e.*, single mass) AAV capsid at varying CSD widths (selected CSD width depicted in the middle). As expected, no unusual spectral effects appear, and CSD width has the simple effect of extending the charge state series. (F–J) Simulations performed with identical to those shown in panels A–D, except using a stochastic population of AAV capsid stoichiometries. Clearly, the spectral appearances between stochastic and homogeneous assemblies diverge greatly, especially at intermediate values of CSD width, where a complex, unresolved distribution is formed at *ca.* 24 000 *m*/*z*. As CSD width gets progressively larger, the apparent signals shift progressively to lower *m*/*z* values due the bias induced by signal concentration. Parameters used: VP1/2/3 ratio 15/15/70; central *m*/*z* position 23 750 *m*/*z*; solvent adduct mass 2000 Da. NB: the idealized appearances in E and J are most similar to the earlier simulations by Wörner *et al.* in Ref. 13.

To assess the diverse spectral appearances depicted in Fig. 1, we next aimed to simulate the spectra based on the stochastic assembly model initially proposed by Wörner *et al.* (Fig. 2, for a more extended description see Fig. S1†).^13^ This model takes into account the 1891 possible co-occurring capsid stoichiometries (based on *n* = 3 different VPs with *k* = 60 subunits total, giving (*k* + *n* − 1)!/(*k*! × (*n* − 1)!) unique combinations), weighted by their statistically-predicted abundances based on the relative subunit (VP1, VP2, VP3) concentrations. Fig. S2† depicts a native mass spectrum simulated in this manner, mirroring the previously reported results of Wörner *et al.* These simulations reproduce the seemingly well-resolved triplet peak appearance of the AAVs as seen in AAV8-1 (Fig. 1A), but clearly do not match the experimental spectral appearances of AAV8-2 (Fig. 1B) or AAV8-3 (Fig. 1C).

A missing piece of the puzzle is that in initial simulations of Wörner *et al.*, and by extension Fig. S2,† an arbitrarily wide CSD for each component spectrum is assumed (*i.e.* substantially larger than the spectral window).^13^ In reality, the CSDs of protein complexes, when electrosprayed under native conditions, are quite narrow (*i.e.* on the order of several charges).^10^ Therefore, we next repeated the spectral simulations, but decreased the CSD widths to better reflect experimentally observed widths. This analysis revealed that the predicted appearances of the AAV native mass spectra are very sensitive to the width of the underlying CSDs (Fig. 2F–J).

**Fig. 3:**
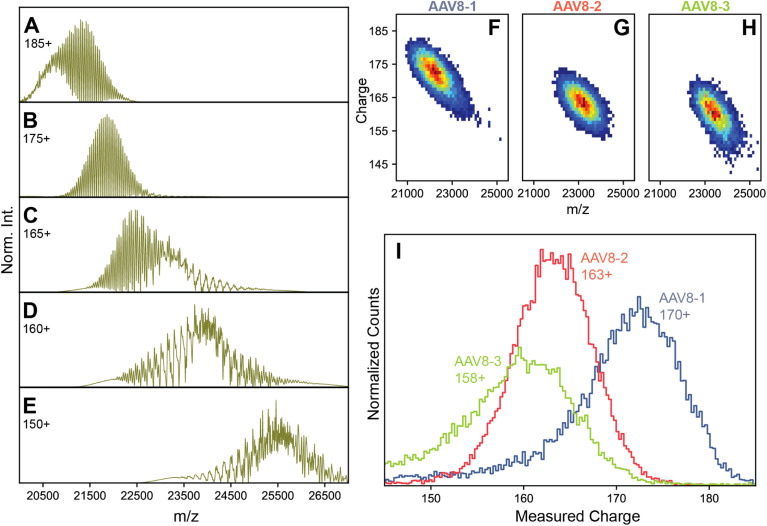
Charge substantially affects spectral appearances of AAVs. (A–E) simulated native mass spectra of an AAV capsid at different average charge values (annotated) but fixed CSD widths of 12. Of the simulated average charge values, the 175^+^ produced a spectrum fully matching the earlier reported “false” triplet appearance. Higher average charge values produce a population of signals that are less resolved, while lower average charge values produce progressively more complex, heterogeneous spectral appearances. At intermediate average charge values (*e.g.* 165^+^), a mixture of both resolved and unresolved signals are observed in different *m*/*z* regions of the spectra. Panels B, C, and D are most similar to the experimental native mass spectra in Fig. 1A–C, respectively. Parameters used: VP1/2/3 ratio 15/15/70. (F–H) Two-dimensional Orbitrap-based CDMS charge *vs. m*/*z* histograms of each AAV8 preparation. The progressively lower average charge (and therefore higher *m*/*z* values) are readily apparent between the preparations. (I) The one-dimensional charge histogram for each AAV8 capsid as measured by Orbitrap-based CDMS. The average charge of each preparation is annotated.

These refined simulations reveal that the appearances of the experimental native mass spectra would change dramatically depending on their occupied *m*/*z* windows. To highlight this effect, we next focused on the effect by producing several simulated spectra at a pre-set CSD width of 12, with an average *z* of 185, 175, 165, 160, and 150, respectively, thereby also shifting the average *m*/*z* positions (Fig. 3A–E). Obviously, this average charge variable also has a profound effect on the appearance of the native MS spectra.

The occupation of different *m*/*z* windows due to differential charging by each of the AAV8 preparations would provide an explanation for their divergent spectral appearances. By their spectral features, our simulations predict that AAV8-1, AAV8-2, and AAV8-3 exhibit progressively lower amount of average charge. Accurately determining the charge values of each AAV8 from these complicated native mass spectra is hampered due to their unresolved distributions (*e.g.*, Fig. 1B and C). To circumvent this limitation, we instead measured the ion charges in each AAV preparation directly using Orbitrap-based CDMS (Fig. 3F–I). The average values of *z*, and by extension *m*/*z*, were indeed found to differ between each preparation, confirming that these *m*/*z* window occupancies are the primary driver of their native mass spectral appearances. Directed by our Orbitrap-based CDMS results, we were able to recapitulate the spectral appearances of all three AAV8 preparations by simulating their stochastic assembly in combination with their ESI charging behaviour (Fig. S4†). These results demonstrate the rich complexity brought by stochastic assemblies for native MS measurements, while highlighting the unique utility of Orbitrap-based CDMS and spectral simulations to assist in interrogating these effects.

### Restricted stochastic heterogeneity in engineered capsids

It is clear that stochastic assembly has a dramatic effect on the spectral appearances of the canonical AAVs in high-resolution native MS, perhaps due to their extremely large number of possible stoichiometries arising from the 3 VP isoforms. In theory, capsids of lower monomer heterogeneity should also assemble stochastically, but the potential effects this intrinsic heterogeneity may have on the capsid distributions and resultant native mass spectra have not yet been explored. To test this in a systematic manner, we next mass analyzed a series of engineered virus-like particles (VLPs) based on the AAV architecture, but with a more precisely defined and reduced heterogeneity. We prepared capsids comprised of only VP1 and VP3 and lacking VP2 (VLP-1) and one comprised of only VP3 (VLP-0). The latter should theoretically generate a completely homogeneous population of capsids, containing 60 copies of only VP3.

**Fig. 4:**
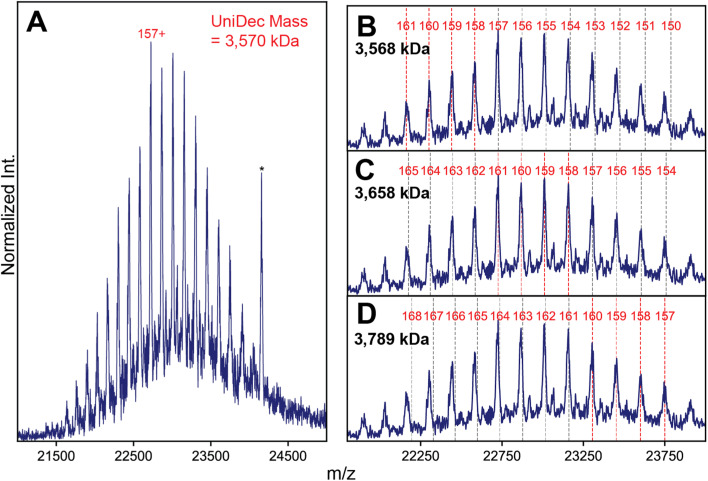
High-resolution native MS of VLP-1 highlights spectral anomalies resulting in an incorrect mass assignment. (A) Native mass spectrum of VLP-1, which lacks VP2 (transient time 256 ms). A seemingly single putative CSD is observed, yielding an apparent mass of 3658 kDa when deconvoluted using UniDec. An electronic noise peak present at *ca.* 24 200 *m*/*z* is annotated by an asterisk (*). (B–D) Alternative charge state assignments for the signals exhibited by VLP-1, highlighting the presence of deviations in the *m*/*z* positions of the observed signals from their predicted positions. The apparent mass derived from each CSD is annotated in each panel.

In contrast to the complex spectral appearances of the more natural AAV capsids, native mass spectra of these engineered capsids yielded simple appearances consistent with a single CSD (Fig. 4A and S5†). Standard charge state inferencing using UniDec^26^ of the VP3 only VLP-0 yielded a mass of 3.589 MDa with charges centered around 160^+^, in excellent agreement with its predicted mass calculated from 60 copies of VP3 (3.588 MDa). For VLP-1, we expected a substantially more heterogeneous spectrum, but observed what seems like a homogeneous, single CSD (Fig. 4A). One could easily interpret this spectral homogeneity as evidence of a homogeneous (*i.e.*, non-stochastic) population, in direct contradiction to the stochastic assembly of the canonical AAVs. Unusually, deconvolution of these ion signals using UniDec yielded a mass of 3570 kDa, which is remarkably even lower than the mass of VLP-0 which contains only the smallest VP isoform.

To try and rationalize the unexpected results of VLP-1, we closely scrutinized the native mass spectrum of VLP-1, and discovered several features present that are atypical for a normal native mass spectrum. Firstly, some of its “charge states” exhibit unusual *m*/*z* positions shifted from their expected values (Fig. 4B). While signals in the lower-*m*/*z* portion of the spectrum agree well with their expected positions, signals at higher *m*/*z* values exhibit progressively larger deviations (*i.e.* they appear at a lower *m*/*z* than expected). This observation is highly unusual in a native mass spectrum, as any charge state-dependent *m*/*z* shifts that one would expect, such as differences in desolvation efficiency, manifest as higher masses at increasing *m*/*z*, opposite of what is observed. Secondly, signals at the center of the distribution seem to be sharpest, with peak broadening observed at both lower and higher *m*/*z* values, again opposite to what is expected from solvation-induced effects. Neither of these effects are observed in the VP3-only particle (Fig. S5†). The impact of these spectral aberrations is most readily apparent when classical charge state inferencing (*e.g.*, by computing charge from the *m*/*z*-spacing between adjacent peaks^11^) is performed on different subsets of the mass spectrum of VLP-1. In theory, charge state inferencing should be insensitive to which specific peaks are chosen for this analysis, as all peaks arise from the same underlying CSD. This is not the case for VLP-1, with apparent masses differing by more than 200 kDa depending on which subset of peaks are utilized for inferencing due to the different extracted charge values (Fig. 4C and D). Despite its deceptively “assignable” appearance, there is no single CSD that adequately recapitulates all the *m*/*z* positions experimentally observed for VLP-1.

**Fig. 5:**
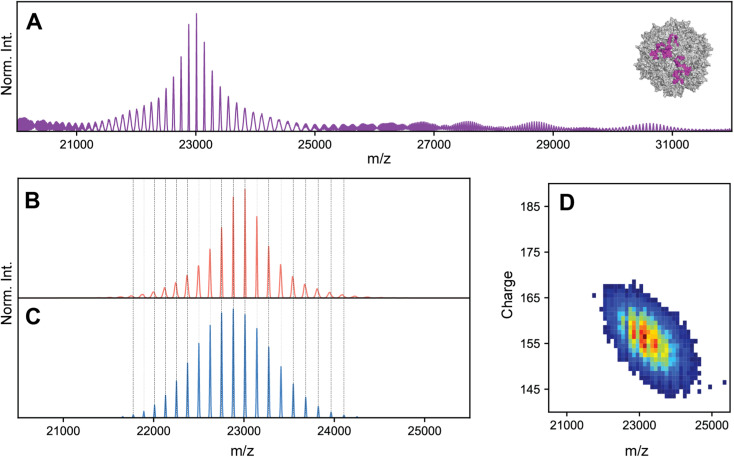
Effects of stochastic assembly on the native mass spectral appearance of VLP-1. (A) Representative simulated native mass spectrum of VLP-1 assuming stochastic assembly, using an arbitrarily large CSD width. Signals are primarily concentrated around 23 000 *m*/*z*. The average VP1 : VP3 ratio used was 1 : 2. (B) Simulated native mass spectrum assuming stochastic assembly, using a finite CSD width. The peak broadening at the extremities of the distribution can be readily observed in the simulated spectrum, as was observed in the experimental mass spectrum in Fig. 4C Simulated native mass spectrum assuming a single, homogeneous assembly, producing a single CSD. Deviations between these *m*/*z* positions and those of the stochastic distribution can be observed (vertical dashed lines). (D) Experimental two-dimensional Orbitrap-based CDMS charge *vs. m*/*z* histograms of VLP-1, confirming that the charging behaviour of its ion population places it in the *m*/*z* region of signal concentration outlined in panel A.

To investigate the possibility of stochastic assembly as the root cause of these spectral anomalies, we simulated the native mass spectrum of VLP-1 assuming a stochastic population of its 61 possible capsid stoichiometries (Fig. 5). Interestingly, the simulated mass spectrum does not produce an unresolved heterogeneous mixture, but instead recapitulates both the anomalous peak broadening and shifted *m*/*z* positions observed in Fig. 4, confirming that VLP-1 is indeed also an ensemble of particle stoichiometries despite its homogeneous spectral appearance. Like the canonical AAVs, this stochastic assembly of VLP-1 yields *m*/*z* windows where signal concentration effects seem to occur, this time primarily centered around 23 000 *m*/*z* (Fig. 5A). Unlike the canonical AAVs however, this signal concentration does not generate a series of triplet peaks, but instead yields signals similar to a homogeneous, albeit distorted, CSD (Fig. 5B). As such, native mass spectra of these systems could be easily misinterpreted as a homogeneous distribution, belying its true heterogeneity (Fig. 5C). Using Orbitrap-based CDMS, we determined that the average ESI charge carried by VLP-1, serendipitously, centers its mass spectral signals directly in the region at *ca.* 23 000 *m*/*z* where signals are expected to be most concentrated (Fig. 5D), facilitating its high-resolution appearance analogously to what occurred for AAV8-1 (Fig. 1A).

**Fig. 6:**
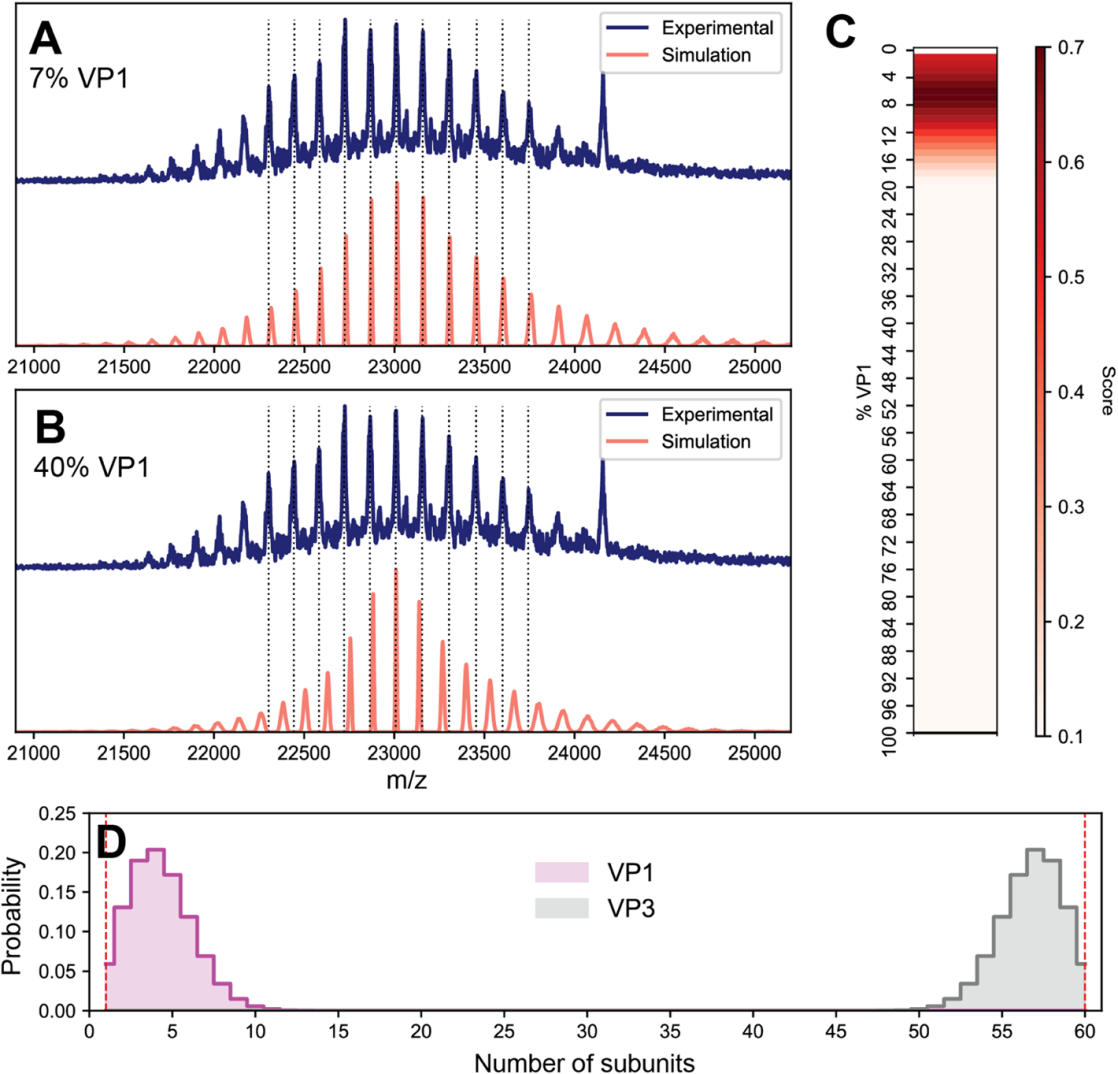
Determining the composition of VLP-1 by spectral simulation scoring following *in silico* VP3_60_ depletion. (A) Comparison of the experimental VLP-1 spectrum (blue) with the best-scoring simulated spectrum (7% VP1, red). Vertical lines denote the experimental peak positions. (B) Representative comparison of a poorly-scoring capsid composition (40% VP1, red). In contrast to panel A, poor alignment of experimental and simulated peaks are observed. (C) Heat map of spectral matching scores as a function of VP1 content. Highest scores are centered around a VP1 content of 7%. (D) Probability distribution of subunit quantities extracted from the stochastic simulations of 7% VP1 as shown in panel A. The *in silico* depletion of VP3_60_ (equivalent to VP1_0_) capsids results in their probabilities to be zero (highlighted as vertical red lines).

Given that the appearance of the native mass spectrum of VLP-1 does not reflect a CSD and thus cannot be directly deconvoluted, how does one properly extract accurate mass information from such a system? To tackle this challenge, we first attempted a spectral simulation matching strategy analogous to that previously employed for AAVs (Fig. 6).^13^ In brief, native mass spectra at different ratios of VP1 and VP3 were computed assuming a stochastic mixture of capsid stoichiometries, and peak positions directly scored against the experimental spectrum. The average mass of the population can subsequently be extracted as the weighted average of the capsid masses from the best matching VP1/VP3 ratio. We found that while the best scoring spectra were localized to fairly low VP1 (<10%) content, there existed several different possible stoichiometries that yielded similar scores, rendering it challenging to accurately identify the correct composition (Fig. S7†).

Despite this ambiguous first result, we reasoned that the native mass spectrum of VLP-1 should encode sufficient information to uniquely identify its capsid content, and so we revisited the assumptions underlying the spectral modeling to target any potential sources of error. So far, it has been assumed that the particle populations reflect a completely stochastic (*i.e.*, unbiased, random) assembly process, which seems to adequately describe the composition of the canonical AAVs.^13^ An important detail regarding VLP-1 is that it was engineered to contain a His-tag specifically inserted in the VP1 monomer which was utilized to purify the capsids *via* a Ni^2+^ affinity column. The purified VLP-1 capsids therefore cannot contain any particles in which VP1 is absent, thereby causing the final VLP-1 capsid preparation to not truly reflect a fully stochastic population. To reflect this in the spectral simulations, we performed an *in silico* depletion by removing the contributions of any capsid missing VP1 (*i.e.* VP3_60_ capsids). This modification will be most pronounced at low proportions of VP1, when the probability of forming VP3-only capsids is substantial. In the spectral matching using these attenuated VLP-1 capsid populations, the best scoring results coalesced into a single value at 7% VP1, yielding an ensemble-averaged mass of approximately 3680 kDa (Fig. 6A), more than 100 kDa larger than the apparent mass obtained *via* the initial naïve charge inferencing, as depicted in Fig. 5A. These results demonstrate not only that even protein complexes that putatively display a single CSD can be a highly heterogeneous ensemble of stoichiometries, but also that applying a naïve deconvolution to the signals can produce erroneous results for their accurate mass. Moreover, this analysis reveals that high-resolution native MS can be sensitive enough to distinguish subtle difference in the distribution of capsid stoichiometries.

### Stochastic assembly beyond viral capsids

Although our results thus far clearly demonstrate that stochastic assembly and average charge state affect the spectral features in high-resolution native mass spectra of AAVs and engineered AAV-like VLPs, we hypothesize that this may also be true for more biomolecular assemblies. Scrutinizing literature and our own work,^1,6^ with hindsight, we feel that some published data should be re-evaluated in the context of our findings. Here, we wish to briefly demonstrate that analogous effects can occur in other assemblies in a sufficient degree to impact accurate mass extraction. As an illustrative example we focused on ferritin, a 24-mer protein complex which plays a role in iron homeostasis by storing up to several thousand iron atoms in its internal cavity.^27–29^ Ferritin without its iron core, apoferritin (apoF), has shown promise as a vehicle for drug delivery due to its capacity to be engineered to *e.g.* encapsulate small drugs^30,31^ or display antigens on its surface for use as a vaccine.^32,33^ In mammalian cells, apoF is normally comprised of two interchangeable monomers of modest homology: a light chain (L, 19.9 kDa) and heavy chain (H, 21.3 kDa). The ratios of L : H can vary depending on the cells or tissue of origin.^34^ The interchangeability of the L and H subunits within the apoF structure led us to hypothesize that apoF could also assemble stochastically, yielding a distribution of cages of variable composition analogous to that seen in AAVs. A few high resolution native mass spectra of mammalian-derived apoF have previously been reported in the literature, all observed and annotated by using a single resolved CSD.^1,35^

**Fig. 7:**
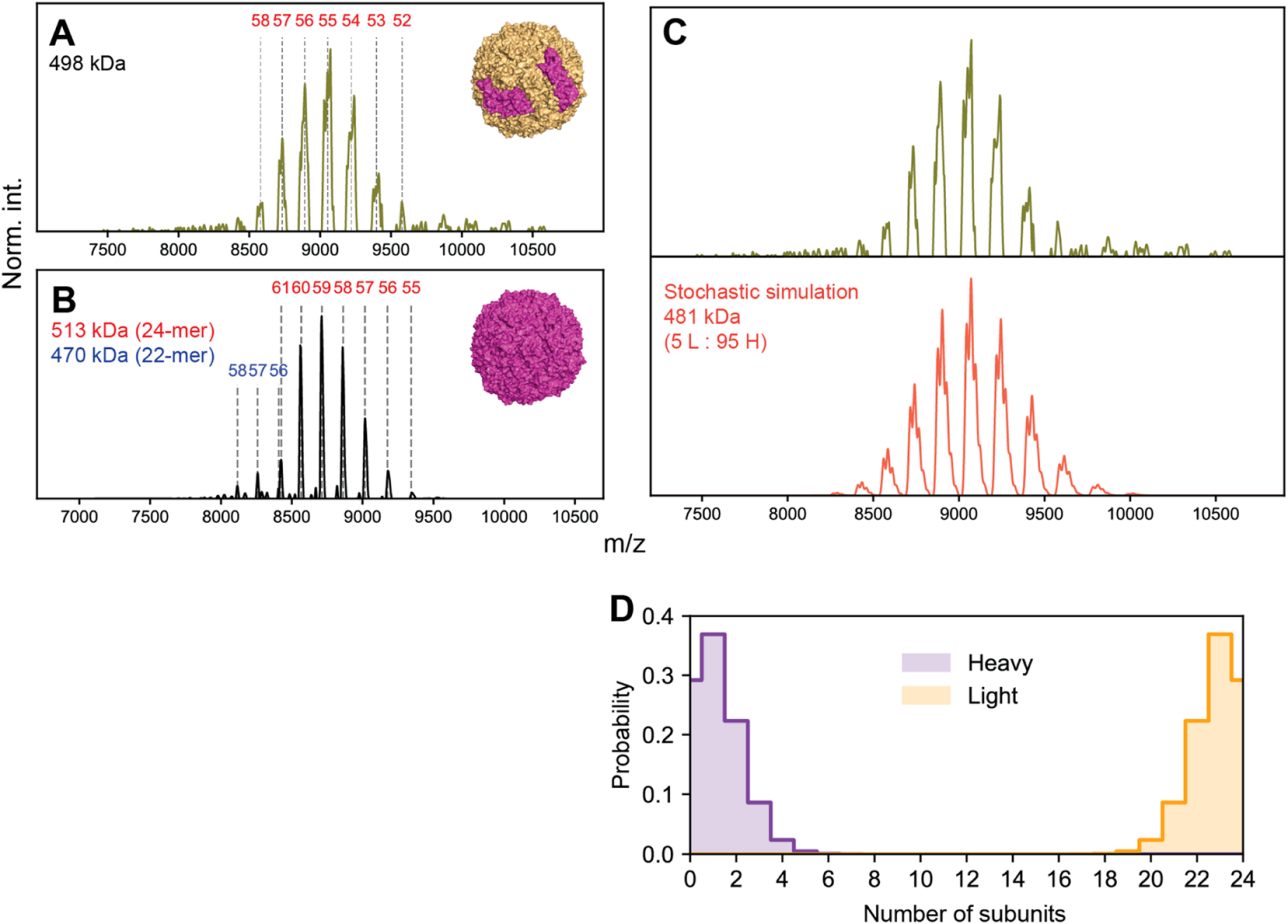
High-resolution native MS of two apoferritin preparations. (A) Experimental native MS of wt-apoF, with putative charge state assignments denoted in red. The apparent mass of wt-apoF obtained from standard inferencing is 498 kDa, approximately 17 kDa larger than its predicted mass. (B) Experimental native MS of r-apoF, which is comprised only of homogeneous H. The spectrum is primarily composed of the expected 24-mer complex (charge states in red). A minor population of incomplete 22-mer complex is also detected (charge states in blue). Both species show excellent agreement with their sequence-predicted masses. (C) Comparison of experimental (top) and simulated (bottom) spectra of wt-apoF. Treating wt-apoF as a stochastic mixture of 5% H and 95% L, corresponding to an average stoichiometry of 1.2 H : 22.8 L, successfully recapitulates the features of the experimental spectrum, such as the broadened peaks and relative positions. (D) Probability distribution of subunit quantities extracted from the stochastic simulations shown in panel C. In the calculated populations, approximately 30% of wt-apoF capsids contain 0 copies of H.

We first performed native MS on a sample of apoF extracted from horse spleen (wt-apoF), where H is present at ∼10%, corresponding to an expected complex stoichiometry of L_22_H_2_ with a mass *ca.* 481 kDa (Fig. 7A).^1,35^ We additionally acquired native mass spectra of a recombinant apoF comprised only of the H-chain (r-apoF), which is expected to be homogeneous (Fig. 7B). For both these assemblies, a single major CSD is seemingly observed. Standard charge state inferencing of r-apoF yields a mass of 513 kDa, in excellent agreement with its sequence-predicted mass of 512 kDa for the recombinant H-chain-only 24-mer. On the other hand, following a similar assignment strategy wt-apoF yields an apparent mass of 498 kDa, substantially higher (17 kDa) than the sequence-predicted mass of ∼481 kDa. Peaks are also noticeably broader in wt-apoF relative to r-apoF. Larger-than-expected mass with broad peaks were also reported in earlier studies of apoF, which was attributed at the time to incomplete desolvation and/or possible presence of residual iron.^35^

When the native mass spectrum of wt-apoF is instead considered within the framework of stochastic heterogeneity, both its abnormal apparent mass and broad peak shape can be completely rationalized (Fig. 7C), in which each apparent charge state is an amalgamation of several, unresolved stoichiometries of different L : H ratios. The mass determined *via* spectral modeling of stochastic heterogeneity yields a mass much closer to expected (481 kDa), without any anomalous adduction (Fig. 7D). Evidently, the presence of these unresolved stoichiometries gives rise to sufficient deviations in the *m*/*z* peak positions to introduce erroneous charge state and mass assignments.

## Concluding remarks

The accurate determination of the composition of biomacromolecular assemblies is critical to their characterization. Here, we provide experimental evidence that several protein complexes may assemble in a stochastic/statistical fashion, such that the final particle populations are substantially more heterogeneous than often considered. Moreover, we determine that the intrinsic heterogeneity of these systems modulates the appearance of their high-resolution native mass spectra in several unexpected ways, with critical implications for the correct spectral interpretation of this important class of macromolecular structures.

We propose that this intrinsic, statistical heterogeneity of biomacromolecular complexes is a generally overlooked phenomenon in native MS. While native MS had previously provided experimental evidence for the stochastic nature behind the assembly of yet another protein complex (the tumor suppressor nucleoside diphosphate kinase hexamer, consisting of NME1/NME2 subunits),^36^ its relatively low molecular weight (∼100 kDa) still allowed for well-resolved individual CSDs, such that each unique stoichiometry could be clearly differentiated. In contrast, for larger oligomeric assemblies where these stoichiometries are unresolved, the spectral signal effects we describe here tend to “mask” the underlying complexity of the system and can be easily mistaken for a homogeneous signal. In a similar vein, commonly employed techniques such as single particle cryoEM rely on averaging over a particle ensemble, such that any structural deviations between individual particles can be challenging or impossible to ascertain. To this effect, virtually all reported structures of both AAVs and (non-recombinant) apoferritin do not resolve the minority isoforms (VP1/2 and H, respectively), despite their expected presence at ∼10%.^37–39^ The interpretative framework using native MS we outline in this work may be unique in its capacity to detect and quantify the heterogeneity of these larger assemblies.

One important implication from our analyses is that for any particular macromolecular complex, if assembled stochastically, “well-resolved” native mass spectra may not ever be generated if to their ESI charging behaviour places them in an unfortunate *m*/*z* window where no signal concentration effects occur. While such results could be naïvely attributed to poor-quality data and/or sample degradation, these spectral appearances would be in fact intrinsic to the heterogeneous particle population and could be fully rationalized by applying the analytical approach we describe in this work. This may explain, for example, the growing discrepancies in the literature of high-resolution native MS appearances of AAVs, which range from seemingly well-resolved^19^ to heterogeneous and featureless.^40,41^ The precise point of transition between these two extremes will depend on not only the average mass and charge of the particle ensemble, but also on for instance the mass differences between distinct subunits and their relative abundances, and thus be different for any given assembly.

For cases less extremely heterogeneous than canonical AAVs, such as VLP-1 or wt-apoF, naïve charge state inferencing of their mass spectra yields masses that can be close to their true mass, but with the possibility of systematic errors. The magnitude of this error is such that it could easily be misattributed as *e.g.* small ligand binding, an alternative complex stoichiometry, and/or incomplete desolvation. In this work, our *a priori* knowledge of the underlying particle composition enabled us to readily identify these heterogeneity-induced artefacts. This may not always be as easily decoupled, *e.g.* in the analysis of protein complexes directly from biological material that may contain endogenous ligands or unknown isoforms.^42,43^ As native MS moves towards the analysis of *in vivo* protein assemblies, it may be the case that any putative masses obtained from these approaches need to be carefully cross-validated to differentiate real mass shifts from stochastic heterogeneity-induced deconvolution errors.

Finally, we wish to emphasize that although we have generally assumed a non-biased, stochastic model for the assemblies of these protein complexes for the sake of simplicity, as we demonstrate for the VP3-depleted VLP-1 this does not necessarily have to be the case. For example, there may be scenarios where incorporation of a certain monomer may destabilize the complex if present in too high abundance, or perhaps where the primary building block is multimeric, such that complexes with a particular monomer count are disfavoured. Generally speaking, any probabilistic model of particle assembly could be invoked, and their native mass spectra simulated and compared with experimental observations. We anticipate that the methodological approach we outline in this work will assist in interrogating the precise compositions of particle assemblies.

## Methods

### AAV8 expression and purification

HEK Viral Production cells (ThermoFisher) were maintained in suspension in Freestyle F17 (ThermoFisher) chemically defined, serum-free media supplemented with 1× Glutamax (Gibco). The cells were cultured in 2 L roller bottles (Greiner) at 37 °C, 7% CO_2_ and 135 rpm agitation in a humidified atmosphere. The plasmids used were pAAV_zsGreen, pAAV_RC8, pAAV_Helper (produced in-house). Cells were seeded into 9 L medium at 0.5 × 10^6^ cells per mL in a wave bioreactor (Cytiva) on a Wave25 platform. 24 hours post-inoculation the culture was triple transfected using PEIMax (Polyplus) and three plasmids at a ratio of 2 : 1.5 : 1. To produce empty AAV particles, the culture were transfected identically except with the absence of the transgene plasmid (pAAV_zsGreen).

Each culture was harvested by batch centrifugation (3800*g* for 15 min at 4 °C) 72 hours post transfection to separate the cell fraction from the supernatant. The resulting cell pellet was freeze-thawed thrice and re-suspended to 10% (w/v) in a lysis buffer containing 5% (v/v) Polysorbate 80 and 20 U mL^−1^ benzonase (Merck). After 1 h incubation at 37 °C, the lysate was clarified by depth filtration followed by sequential filtration through 0.8 and 0.45 μm filters. The clarified lysate was pooled with filtered supernatant and concentrated by TFF on an Ultracel Pellicon 2, 300 kDa MWCO cassette (Merck) before loading onto a pre-equilibrated POROS CaptureSelect AAVX affinity column (Thermo Fisher) at 1 × 10^13^ vg per mL of resin. After re-equilibration, AAV was eluted with a low-pH buffer and immediately neutralised with 1 M tris base. Neutralised eluate was concentrated and buffer exchanged into PBS using an Amicon Ultra, 100 kDa MWCO concentrator (Merck) before storage at −80 °C.

### VLP expression and purification

The protein sequences used in the design of constructs for expression of AAV8 VP1, VP3 and Assembly-Activating Protein (AAP) proteins were derived from UniProt entry Q8JQF8. A 6×His tag was placed within an exposed loop of the VP1 protein, VP3 was left untagged and AAP was tagged at the N-terminus with the Flag tag. The VP1 coding region followed by an IRES and the AAP coding region was cloned into the pDEST12.2 OriP (Thermo Fisher) plasmid. The VP3 protein coding region was cloned into a separate pDEST12.2 OriP (Thermo Fisher) plasmid. The constructs were expressed in suspension Chinese Hamster Ovary (CHO) cells following transfection with a 1 : 2 molar ratio of VP1/AAP plasmid to VP3 plasmid and capsids were purified from the conditioned media using standard immobilized metal affinity chromatography, anion exchange and size exclusion chromatography purification.

For the VP3-only capsid, the same sequences as described above were used to create a pDEST12.2 OriP vector containing the AAV8 VP3 coding region followed by an IRES and the AAP coding region. HEK293/T17 cells were transfected with the construct and cultured for 70 h. The virus like particles (VLPs) were harvested from the cells by sonication and purified by flocculation by following the protocol of Potter *et.al.* using conditions described for AAV8.^44^ The purified AAV8 VP3 VLPs were concentrated and buffer exchanged into DPBS + 0.001% Pluronic F68 (Sigma P1300).

### Apoferritin

Horse spleen apoferritin was purchased from Millipore Sigma (A3660). Recombinant heavy chain-only apoferritin was purchased from ThermoFisher Scientific (A51362).

### Native mass spectrometry

For AAVs, samples were buffer exchanged into 75 mM ammonium acetate pH 7.5 by serial dilution using 50 kDa Amicon-0.5 MWCO centrifuge filter units (Sigma-Aldrich). For each sample, approximately 30 μL was centrifuged for 6 cycles of 10 minutes each at 9000 G. For ferritin, samples were buffer exchanged into 150 mM ammonium acetate pH 7.5 by serial dilution using 50 kDa Amicon-0.5 MWCO centrifuge filter units (Sigma-Aldrich). Each sample was centrifuged for 9 cycles of 10 minutes each at 10 000 G.

AAV8 and VLP native MS measurements were performed on an Orbitrap Q Exactive UHMR mass spectrometer (Thermo Fisher Scientific) using xenon as collision gas. Apoferritin native MS measurements were performed on an Orbitrap Q Exactive EMR mass spectrometer (Thermo Fisher Scientific) using nitrogen as collision gas. For each measurement, approximately 1.5 μL of sample was loaded into a gold-coated borosilicate capillary (prepared in-house), and electrosprayed in positive ion mode.

Samples for standard, ensemble native MS were typically analyzed without further dilution following buffer exchange. For Orbitrap-based CDMS measurements, AAV samples were diluted by *ca.* 100-fold prior to measurement. Ion injection times were manually attenuated to reach the single particle detection regime. A tabulation of typical instrument parameters for both native MS and Orbitrap-based CDMS can be found in Table S2.†

### MS data processing and analysis

Single particle CDMS data was processed in Python as previously described using an intensity-to-charge calibration factor of 12.521.^36^ Simulations of the native mass spectra were performed in Python utilizing the method provided in Wörner *et al.* for simulation of AAV mass spectra, with minor modifications to allow simulation of non-AAV based particles.^35^ Briefly, the theoretical probability distribution of the protein assembly is calculated *via* a stochastic assembly model. For any specified monomer isoform ratio, a simulated native mass spectrum is constructed by the probability-weighted average mass spectrum composed of the spectra from all possible particle stoichiometries. For the simulation of VLP-1, the contribution of VP3_60_ capsids were removed from the population, and probabilities were re-normalized. Unless otherwise noted, the charge state distribution of the simulated mass spectra were chosen to best reflect the experimental data. Molecular weights of AAV VP isoforms were obtained by intact protein denaturing LC/MS (data not shown). For apoferritin, monomer masses were calculated from available sequences. Charge state deconvolution was performed using either UniDec^26^ or by manual analysis of neighbouring *m*/*z* peaks.^11^ Scoring of simulated *versus* experimental native mass spectra was performed as previously described for AAVs by Wörner *et.al.*^13^

## Data availability

A detailed description of experimental parameters utilized to generate both native MS and CDMS data sets are provided in the ESI.†

## Author contributions

Project conception: VY, and AJRH. AAV sample production: MD, JCS, FC, and AR. AAV sample quality control: PWAD, AH, SS, and MD. Mass spectrometry, data analysis and simulations: VY. Supervision: NJB, CD, JS, AJRH. Writing: VY and AJRH with input from all other authors.

## Conflicts of interest

PWAD, JCS, FC, AR, AH, SS, MD, CLD and NJB are employees of AstraZeneca, a company with an interest in developing adeno-associated viruses for gene-delivery.

## Supplementary Material

SC-014-D3SC03228K-s001
