# Supplementary material for: Stochastic assembly of biomacromolecular complexes: impact and implications on charge interpretation in native mass spectrometry
Source: Chem Sci. 2023 Aug 17;14(35):9316–27. doi: 10.1039/d3sc03228k (PMC10498669; doi:10.1039/d3sc03228k)
Supplement: SC-014-D3SC03228K-s001 [file SC-014-D3SC03228K-s001.pdf]

Electronic Supplementary Material (ESI) for Chemical Science.
This journal is © The Royal Society of Chemistry 2023

SUPPORTING INFORMATION

Stochastic Assembly of Biomacromolecular Complexes: Impact and Implications on Charge Interpretation in Native Mass Spectrometry

Victor Yin[1,2], Paul W.A. Devine[3], Janet C. Saunders[4], Arjan Barendregt[1,2], Fiona Cusdin[4], Alexandra Ristani[4], Alistair Hines[3], Sam Shepherd[3], Marcin Dembek[5], Claire L. Dobson[4], Joost Snijder[1,2], Nicholas J. Bond[3], Albert J.R. Heck[1,2,#]

[1]*Biomolecular Mass Spectrometry and Proteomics, Bijvoet Center for Biomolecular Research and Utrecht Institute for Pharmaceutical Sciences, Utrecht University, Padualaan 8, 3584 CH, Utrecht, The Netherlands*

[2]*Netherlands Proteomics Center, Padualaan 8, 3584 CH, Utrecht, The Netherlands*

[3]*Analytical Sciences, Biopharmaceutical Development, R & D, AstraZeneca, Granta Park, Cambridge, U.K.*

[4]*In Vivo Expressed Biologics, Discovery Sciences, R & D, AstraZeneca, Granta Park, Cambridge, U.K.*

[5]*Purification Process Sciences, Biopharmaceutical Development, R & D, AstraZeneca, Granta Park, Cambridge, U.K.*

This file contains:

Supporting Figures

Figure S1: Scheme depicting the mass spectral modeling workflow utilized in this work.

Figure S2: Full spectral window of a simulated AAV capsid using an arbitrarily large CSD width approach as reported in ref [[13]].

Figure S3: nsTEM images of each AAV8 preparation.

Figure S4. Comparison of experimental (black) vs. simulated native MS (red) of AAV8 preparations utilizing a stochastic assembly model.

Figure S5. Native MS of VLP-0.

Figure S6. nsTEM of VLP-1.

Figure S7: Spectral scoring of VLP-1 prior to depletion of VP1-absent capsid stoichiometries.

Supporting Tables

Table S1: Attributes of the four AAV8 preparations used in this work.

Table S2: Typical instrument parameters used for native MS and CD-MS measurements on an Orbitrap UHMR mass spectrometer.

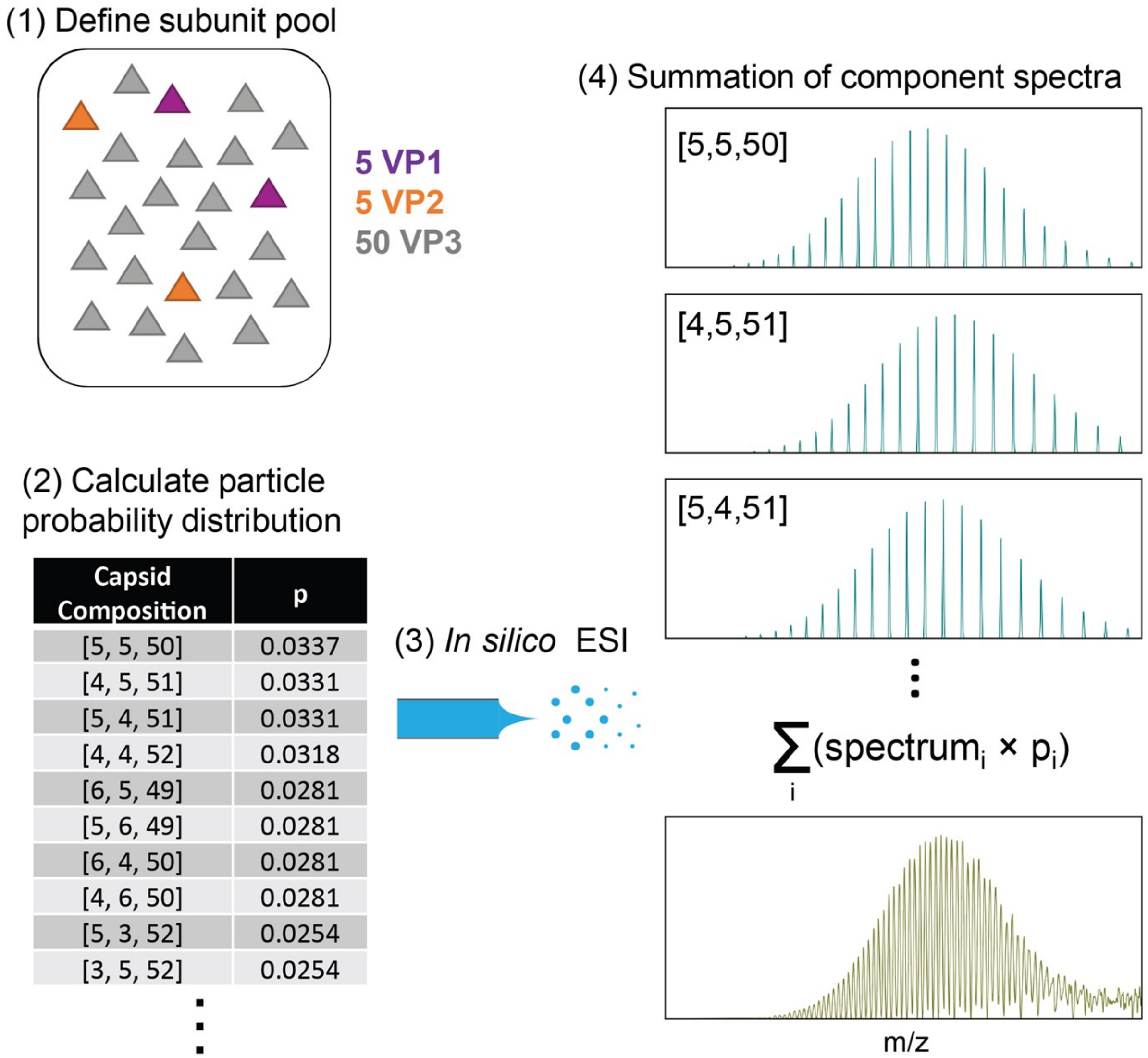

Figure S1: Simplified scheme depicting the mass spectral modeling workflow utilized in this work. (1) An initial pool of distinct monomeric subunits with associated relative ratios are defined. (2) This information is utilized to construct a probability distribution containing every unique particle composition, assuming a stochastic (*i.e.* random) assembly model. In the case of canonical AAVs, this constitutes 1,891 possible combinations. (3) For a given particle composition, the mass is calculated from its constituent monomers. A native mass spectrum for each composition is then generated ("*in silico* ESI") by assigning the particle a pre-defined charge state distribution, spectral resolution, degree of solvation, etc., as described in ref [13]. (4) The final simulated native mass spectrum of the particle ensemble is then produced by the weighted average summation of all particle spectra generated in step 3.

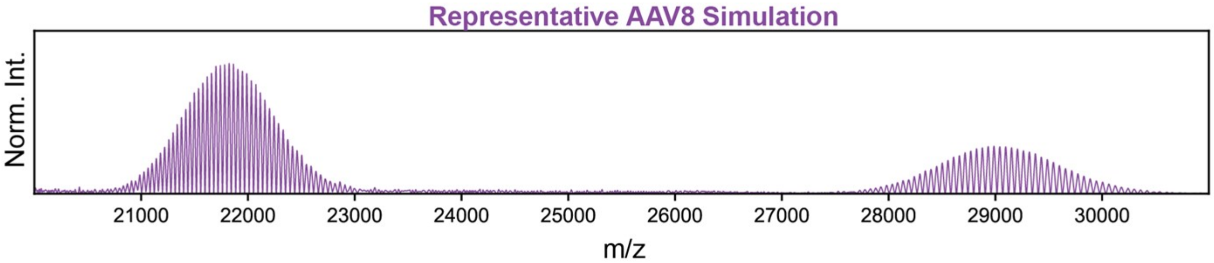

Figure S2: Full spectral window of a simulated AAV capsid using an arbitrarily large CSD width approach as reported in ref [13]. Under these conditions two regions of signal concentration are observed exhibiting the classic triplet appearances: a main population centered at ca. 22,000 *m/z*, and a second theoretical population centered at ca. 29,000 *m/z*, which is not experimentally observed due to its unrealistically low charge.

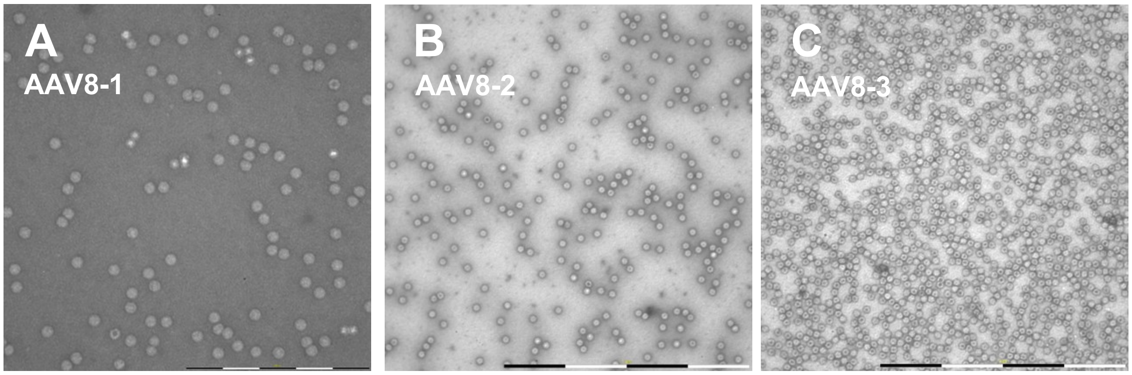

Figure S3: nsTEM images of each AAV8 preparation studied. Carbon-coated copper 400 mesh grids (Ted Pella, CA, USA) were glow discharged for 1 minute using a PELCO easiGlow system (Ted Pella, CA, USA), prior to sample preparation. A 10 μL droplet of sample was pipetted onto parafilm and the prepared grids placed on top of the droplet, allowing adhering of the sample for 60 seconds. Excess sample was removed using Whatman filter paper, before washing the grid on two consecutive 20 μL droplets of ddH2O, before excess water was again removed by blotting against filter paper. Finally, the sample embedded grids were then stained by placing the grids on to a 20 μL droplet of 1% uranyl acetate for 30 seconds, where excess stain was then removed by blotting with filter paper. The prepared grids were then visualised and examined using a Vironova miniTEM system (Vironova, Sweden). Images were collected at a range of magnifications, with images for quantitation being captured at 1 and 1.5 μm field of view (FOV). Scale bars: 500 nm for A, 1000 nm for B and C. Intact capsid particles are readily observed in all preparations.

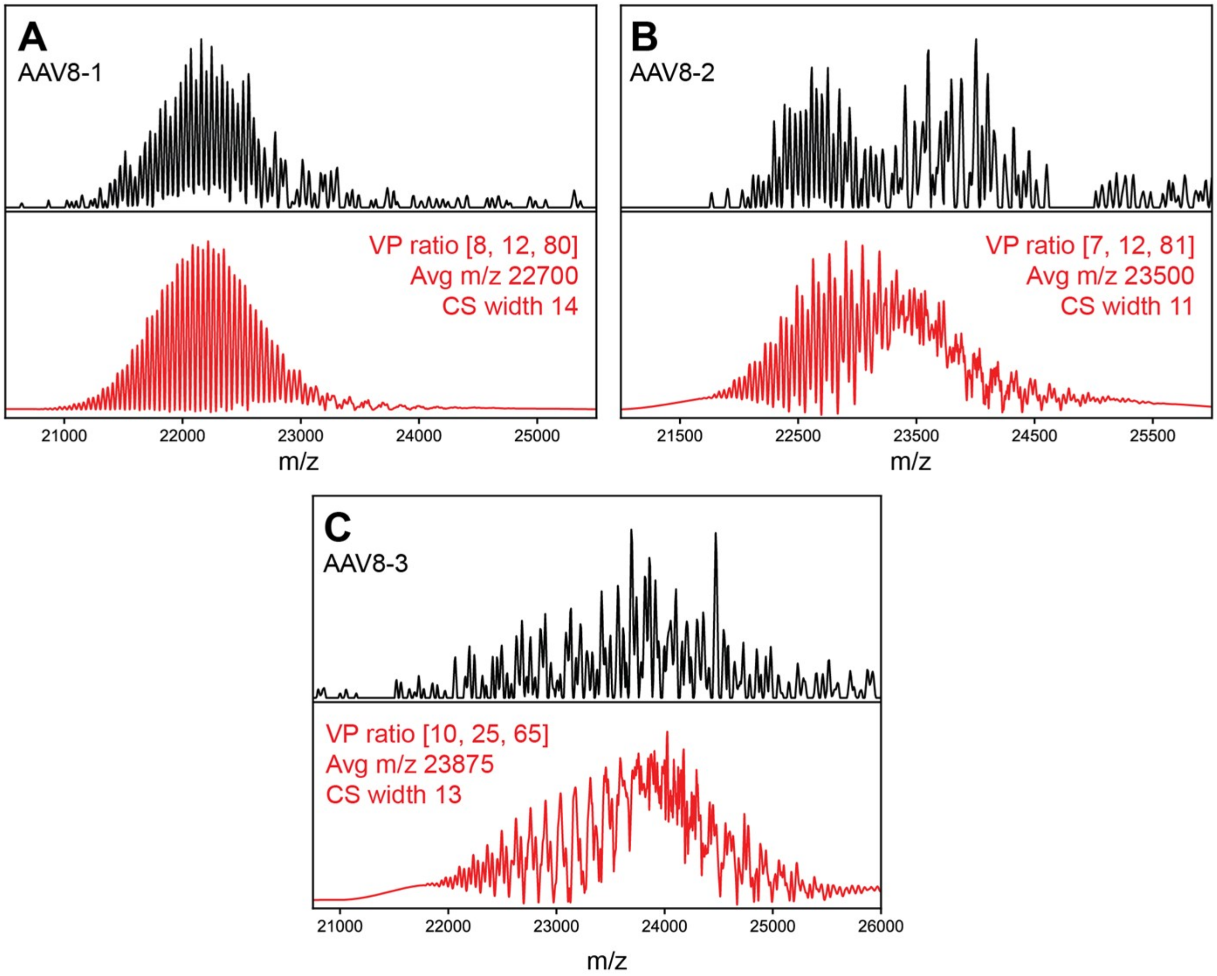

Figure S4. Comparison of experimental (black) vs. simulated native MS (red) of AAV8 preparations utilizing a stochastic assembly model. (A) AAV8-1. (B) AAV8-2. (C) AAV8-3. Key parameters for the simulated spectra are denoted in each panel.

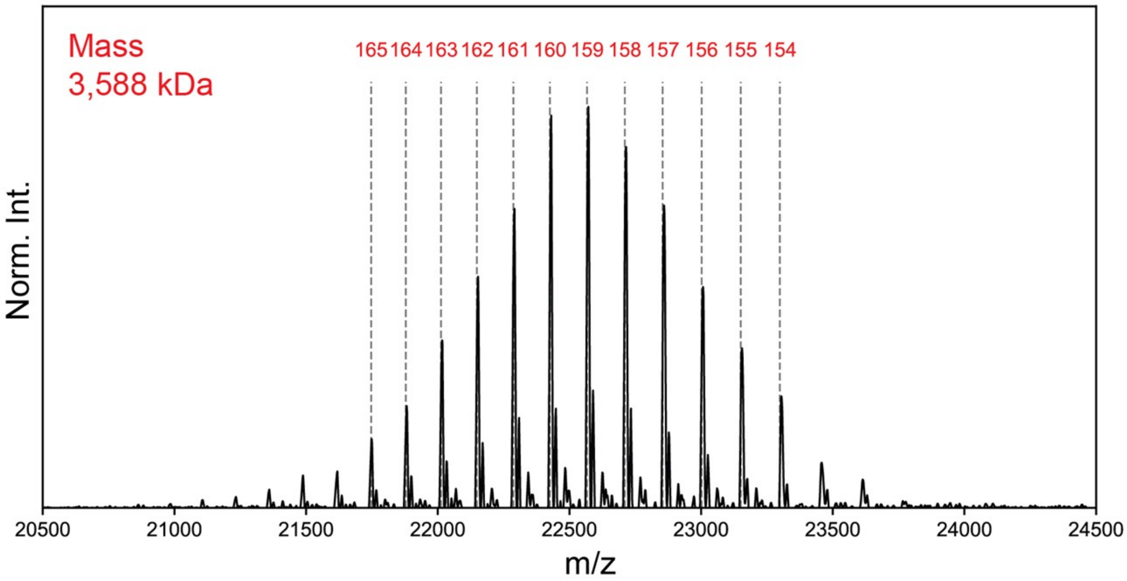

Figure S5. Native MS of VLP-0. A single major CSD is observed, with excellent agreement between theoretical (vertical dashed lines) and experimental peak positions. The deconvoluted charge of each peak is shown in red.

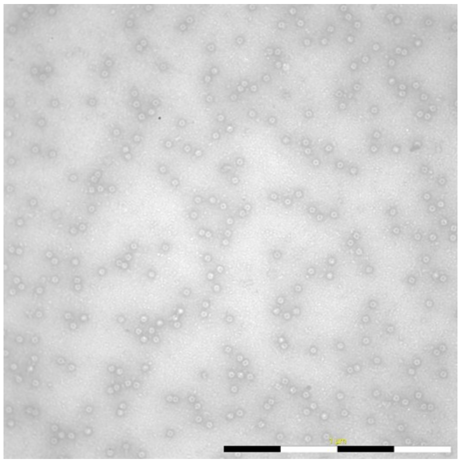

Figure S6. nsTEM of VLP-1. Scale bar: 1000 nm. Intact capsids are readily observed.

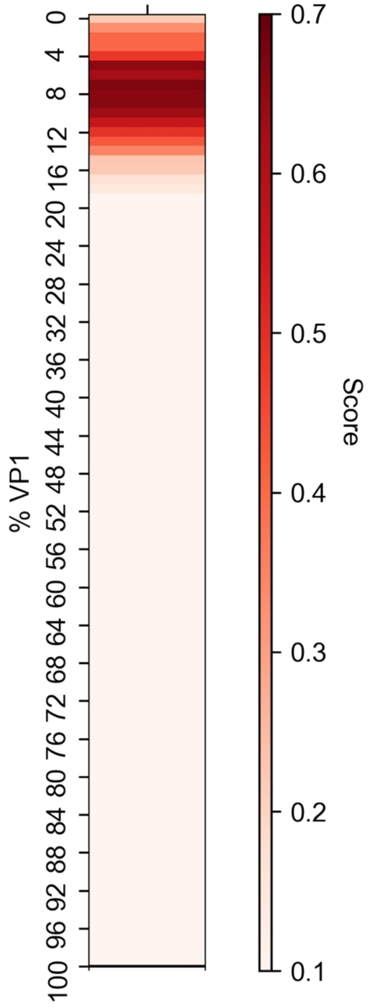

Figure S7: Spectral scoring of VLP-1 prior to depletion of VP1-absent capsid stoichiometries. Although stoichiometries are concentrated at low values of % VP1, there exists several values of maximal probability (e.g. 5% VP1, 8% VP1, etc.).

Sample Name	Transfection Format	Transgene		VP Content		
		Identity	%Full	%VP1	%VP2	%VP3
**AAV8-0**	**Double**	**/**	**0**	**8**	**12**	**80**
**AAV8-1**	**Double**	**/**	**0**	**7**	**12**	**82**
**AAV8-2**	**Triple**	**ZsGreen**	**16**	**10**	**25**	**65**

Table S1: Attributes of the four AAV8 preparations used in this work. The empty to full (E/F) content of each preparation was initially assessed by ion exchange chromatography (IEC), while the relative monomer ratios of each VP isoform were assayed by capillary gel electrophoresis (CGE).

	(Ensemble) Native MS	CDMS
Capillary voltage (kV)	1.30	
m/z range	10000 – 40000	
Ion injection time (ms)	30	10 - 1000
Transient time (ms)	32	1024
Microscans	10	1
Averaging	1000	0
Noise threshold	3.64	0
In-source trapping voltage (V)	-75	
HCD voltage (V)	150	
Trap gas setting	4.0	1.0 – 2.5
UHV readout (1e-10 mbar)	ca. 6 - 8	ca. 1.4 to 4
Collision gas	Xenon	
Injection flatapole (V)	10	
Inter-flatapole lens (V)	10	
Bent flatapole (V)	4	
Transfer multipole (V)	4	
Ion transfer target	High *m/z*	
Detector optimization	High *m/z*	

Table S2: Typical instrument parameters used for native MS and CDMS measurements on an Orbitrap UHMR mass spectrometer.
